# Visceral adiposity indicators as predictors of metabolic syndrome in postmenopausal women

**DOI:** 10.4274/tjod.galenos.2019.62558

**Published:** 2019-10-10

**Authors:** Gökçe Anık İlhan, Begüm Yıldızhan

**Affiliations:** 1 Marmara University Faculty of Medicine, Department of Obstetrics and Gynecology, İstanbul, Turkey

**Keywords:** Menopause, metabolic syndrome, lipid accumulation product, visceral obesity

## Abstract

**Objective:**

The aim of the present study was to evaluate the importance of visceral adiposity indicators on metabolic parameters in postmenopausal women.

**Materials and Methods:**

This cross-sectional study included 200 postmenopausal subjects. Postmenopausal women were divided into two groups based on the presence of metabolic syndrome (MetS) as MetS+ and MetS-. Comparisons of clinical and metabolic characteristics were performed between the groups.

**Results:**

The current study included 200 postmenopausal women and 63 subjects were diagnosed as having MetS. Postmenopausal women with MetS demonstrated significantly higher values with respect to systolic and diastolic blood pressures, body mass index (BMI), waist-hip ratio (WHR), triglyceride (TG), lipid ratios, Homeostasis Model Assessment Insulin Resistance (HOMA) index, TG glucose (TyG), Visceral Adiposity Index (VAI), and lipid accumulation product (LAP) when compared with women without MetS. Correlation analyses showed that LAP and VAI were positively correlated with waist circumference, WHR, BMI, TG, lipid ratios, TyG and HOMA index, and with each other. LAP was also positively correlated with blood pressures.

**Conclusion:**

Visceral adiposity indicators may be useful as predictors of MetS in postmenopausal women.

**PRECIS:** Visceral adiposity indicators may be useful in the early detection of metabolic syndrome in postmenopausal women.

## Introduction

Obesity is a major risk factor for many conditions including metabolic syndrome (MetS) and cardiovascular disease (CVD), and also is a leading avoidable cause of death worldwide^(1,2)^. MetS, a cluster of conditions including abdominal obesity, hypertension, hyperglycemia, and dyslipidemia, serves as a risk factor for type 2 diabetes mellitus (T2DM) and CVD, and is becoming a serious health problem due to the rising trend in the prevalence of obesity worldwide^(1,3)^. Insulin resistance (IR) is also determined as a hallmark feature and a major underlying mechanism of the syndrome^(4,5,6)^. Abdominal obesity, rather than general obesity, is linked to IR with higher risks of MetS and CVD in postmenopausal women^(7)^. Lipid accumulation product (LAP) and Visceral Adiposity Index (VAI) are clinical markers of visceral obesity and have been proposed as simple, novel metabolic indices, that combine anthropometric parameters and metabolic variables as effective markers that have reliable accurracy for predicting MetS^(8,9)^. The triglyceride glucose (TyG) index, a simple measure that combines fasting plasma glucose and triglyceride (TG), is also determined as a good marker for identifying individuals with IR and MetS^(9,10)^. A recent study emphasized the importance of menopausal status on the predictive value of LAP and VAI for MetS, and further studies are recommended; special attention is suggested while applying these markers in women of menopausal transition^(11)^. In another study, LAP and VAI were also found to be effective markers for identifying the metabolically obese, normal-weight individuals who are predisposed to diabetes and CVD development^(12)^. In a recent meta-analysis, the pooled prevalence of MetS was found as 37.17% among postmenopausal women^(13)^. MetS was also found to be more prevalent in postmenopausal women compared with premenopausal women^(13)^. Additionally, in another meta-analysis, it was also suggested that almost all MetS-associated components except high-density lipoprotein cholesterol (HDL-C) were unfavorably changed after menopause^(14)^. Early recognition of high-risk individuals is important because MetS is a cluster of risk factors for CVD and diabetes, and menopause is associated with an increased risk for MetS^(13,14)^. Simple and reliable indicators for the early detection of metabolic disturbances in postmenopausal women may be beneficial in clinical practice. The current study evaluated the importance of visceral adiposity indicators on metabolic parameters in postmenopausal women.

## Materials and Methods

Two hundred postmenopausal women who attended Marmara University Outpatient Clinics were included in this study after obtaining written informed consent. The study protocol was approved by the Ethics Committee of Marmara University (approval number: 09.2018.039). Subjects with systemic disease, malignancy or those using any medications were excluded from the study. Body mass index (BMI) was calculated after obtaining the weight and height measurements of the subjects. Waist (WC) and hip circumferences were measured and WC-to-hip ratios (WHR) were recorded. This cross-sectional study was approved by the ethics committee of the university and conducted in accordance with the Helsinki Declaration. Participants were grouped according to the absence or presence of MetS diagnosed according to the National Cholesterol Education Program Adult Treatment Panel III  criteria^(15)^. The diagnosis of MetS was made depending on the presence of at least 3 of the following parameters: abdominal obesity (WC ≥88 cm), elevated TG (≥150 mg/dL), reduced HDL-C (<50 mg/dL) elevated blood pressure (≥130/≥85 mmHg), and elevated fasting plasma glucose (≥110 mg/dL)^(15)^. In addition to the clinical and biochemical evaluation of the postmenopausal subjects, by using fasting insulin and glucose results, the Quantitative Insulin Sensitivity Check index (QUICKI), Homeostasis Model Assessment IR index (HOMA-IR) and fasting glucose-insulin ratio (FGIR) were calculated by using the following formula: HOMA-IR=fasting insulin (µU/L) x fasting glucose (mmoL/L)/22.5 and QUICKI=1/[log fasting insulin (µU/mL) + log fasting glucose (mg/dL)] and (FGIR)= fasting glucose (mg/dL)/fasting insulin (mIU/mL). TyG indices were calculated based on the formula: ln [fasting TG (mg/dL) x fasting plasma glucose (mg/dL)/2]^(16)^. In addition to traditional lipid ratios [TG/HDL-C, total cholesterol (TC)/HDL-C, low density lipoprotein (LDL)-C/HDL-C], calculations of VAI and LAP were also determined by using established formulae from previous studies:

VAI = [WC/36.58 + (1.89 × BMI)] × (TG/0.81) × (1.52/HDL-C)^(17)^

LAP = [WC (cm) – 58] × [TG (mmol/L)]^(18)^.

### Statistical Analysis

Statistical analyses were performed using the SPSS version 20.0 software package and comparisons of baseline demographic, biochemical, and metabolic characteristics were performed between the groups using Student’s t-test. Continuous variables are described as mean and standard deviation (SD) (Table 1). P<0.05 was considered statistically significant. Pearson correlation analyses were performed between VAI and LAP and cardiometabolic features in postmenopausal women (Table 2). Receiver operating curve (ROC) analysis of VAI, LAP, and TyG was performed for the prediction of MetS.

## Results

The baseline demographic, biochemical, and metabolic characteristics of the groups are described in Table 1. The current study included 200 postmenopausal women and 63 subjects were diagnosed as having MetS. Age, LDL-C, and TC levels were similar between the groups. Postmenopausal women with MetS demonstrated significantly higher values with respect to systolic and diastolic blood pressures, BMI, WHR, TG, lipid ratios, HOMA index, TyG, VAI, and LAP when compared with those without MetS. HDL-C, FGIR, and QUICKI were found to be lower in the MetS+ group (Table 1). Correlation analyses showed that LAP and VAI were positively correlated with WC, WHR, BMI, TG, lipid ratios, TyG, and HOMA index, and with each other. LAP was also positively correlated with blood pressures. Correlation analyses also showed that LAP and VAI were negatively correlated with HDL-C, FGIR, and QUICKI in postmenopausal women (Table 2). ROC analysis of visceral adiposity indicators in predicting MetS was performed, which demonstrated 89% sensitivity and 80% specificity of VAI at an optimal cut-off level of 2.04 [area under the curve (AUC) 0.88; 95% confidence interval (CI)=0.83-0.94]. The sensitivity and specificity for LAP was 84% and 78% at a cut-off level of 54.09 (AUC=0.88; 95% CI=0.82-0.93). The TyG index showed 81% sensitivity and 69% specificity at the optimal cut-off level of 8.56 (AUC=0.87; 95% CI=0.81-0.93) in predicting MetS in postmenopausal women.

## Discussion

Modern lifestyle changes, decreased physical activities, and concomitant increase in obesity subsequently result in a rise in the prevalence of MetS, a condition that affects the morbidity and mortality of older women, with an increased risk for CVD and T2DM^(11,19)^. Postmenopausal women merit special attention because they have an increase in central adiposity that contributes to the development of IR and dyslipidemia, which are also components of a cluster of metabolic abnormalities that increases the risk of T2DM and CVD^(11,20,21)^. The detection of postmenopausal women with a high cardiometabolic risk may aid in the implementation of early lifestyle changes and treatment strategies for future CVD risks. Two novel markers of visceral obesity, VAI and LAP, have been regarded as reliable, simple clinical markers and indicators of MetS in the older people^(8,9)^. In a recent study, the AUC of these markers were found to be different in postmenopausal women than in premenopausal women, and it was suggested that studies evaluating the predictive value of these clinical indicators in postmenopausal women were needed because most studies evaluating these indices were performed in the general population^(11)^. In our study, the AUC of both LAP and VAI was 0.88, in accordance with the study by Lee et al.,^(11) ^which stated that the AUC of both LAP and VAI was 0.89 in postmenopausal women. In a recent meta-analysis, it was reported that the pooled prevalence of MetS among postmenopausal women was 37.17%, ranging from 13.60% to 46% with an overall odds ratio 3.54 times higher than in premenopausal women^(13)^. In our study, the prevalence of MetS was 31.5% in postmenopausal women. Considering the increase in life expectancy and high prevalence of MetS among postmenopausal women, simple and reliable clinical markers to predict metabolic disturbances may be helpful to allow early intervention and to reduce future related complications such as CVD and T2DM. Won et al.^(22)^ found that the TyG index was associated with arterial stiffness in the healthy population and also reported that the prevalence of MetS and diabetes significantly increased with increasing TyG indeces. In a recent study, both MetS as an entity per se and its individual features were found to be significantly associated with subclinical atherosclerosis in postmenopausal women independently of traditional cardiovascular risk factors^(23)^. The TyG index was found to be associated with carotid atherosclerosis and was suggested as a useful marker for identifying high-risk women in the normal-weight postmenopausal population. Additionally, the TyG index was also found to be strongly correlated with HOMA-IR and was suggested as a surrogate index of IR in postmenopausal women^(24)^. Maturana et al.^(25)^ reported LAP as a suitable method to screen for cardiovascular risk in postmenopausal women. Wehr et al.^(26)^ demonstrated an association of LAP levels with T2DM and suggested that high LAP levels were associated with increased mortality in postmenopausal women. A recent study showed that LAP, VAI, and TyG were reliable surrogate markers in identifying MetS in a population aged ≥40 years^(9)^. LAP and VAI were both determined as significant markers to predict the presence and severity of MetS; however, further studies were recommended to apply these markers in clinical practice and to determine appropriate cut-off values for each index in the postmenopausal group^(11)^. In our study, we found significantly higher values for lipid ratios, HOMA-IR, TyG, LAP, and VAI indexes in postmenopausal women with MetS. LAP and VAI were both found to be positively correlated with each other and with BMI, WHR, TG, TyG index, HOMA index, and lipid ratios, and negatively correlated with HDL-C, FGIR, and QUICKI. LAP and VAI were both found to have strong and reliable accuracy for the prediction of MetS in postmenopausal women.

### Study Limitations

Considering the small sample size as a limitation of our study, further studies with larger samples are needed to assess the predictive value of visceral adiposity indicators in identifying MetS in the postmenopausal group. A premenopausal group was not included, which is also a limitation of our study.

## Conclusion

The present study showed that visceral adiposity indicators might be promising in the early detection of MetS in postmenopausal women. Early detection of subjects that are candidates for high cardiometabolic risk is essential, and with regard to the difficulties in assessing cardiovascular risk using traditional measures in postmenopausal women^(27)^, visceral adiposity indicators may be effective for critical primary prevention strategies for subsequent cardiometabolic risks in a woman’s life span.

## Figures and Tables

**Table 1:**
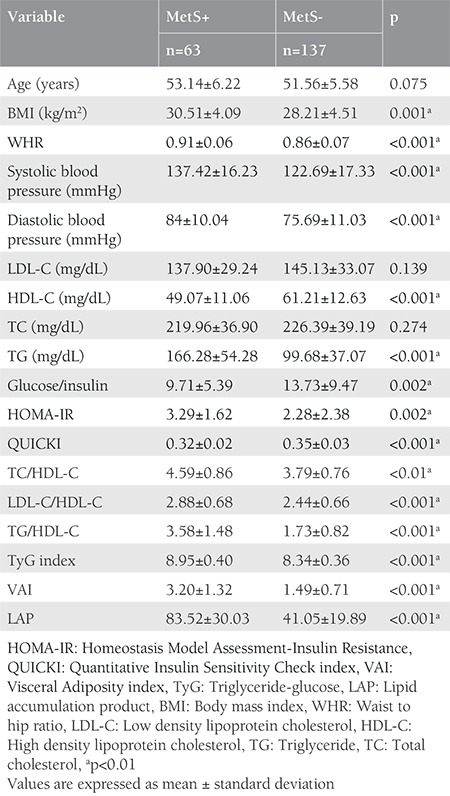
Demographic, biochemical and metabolic characteristics of groups

**Table 2:**
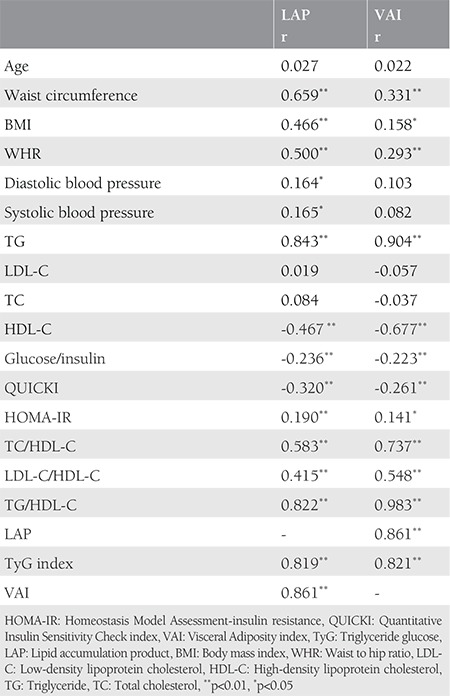
The correlations between lipid accumulation product and Visceral Adiposity Index and cardiometabolic variables in postmenopausal women

## References

[1] Sherling DH, Perumareddi P, Hennekens CH (2017). Metabolic syndrome. J Cardiovasc Pharmacol Ther.

[2] Hennekens CH, Andreotti F (2013). Leading avoidable cause of premature deaths worldwide: case for obesity. Am J Med.

[3] Xu H, Li X, Adams H, Kubena K, Guo S (2018). Etiology of metabolic syndrome and dietary intervention. Int J Mol Sci.

[4] Zimmet P, Shaw J, IDF Epidemiology Task Force Consensus Group (2005). The metabolic syndrome-a new worldwide definition. Lancet.

[5] Guo S (2014). Insulin signaling, resistance, and metabolic syndrome: Insights from mouse models into disease mechanisms. J Endocrinol.

[6] Bonora BM, Marescotti M, Marcuzzo G, Avogaro A, Fadini GP (2015). Synergistic interactions among metabolic syndrome components and homeostasis model assessment of insulin resistance in a middleaged general population over time. Metab Syndr Relat Disord.

[7] Goh VHH, Hart WG (2018). Excess fat in the abdomen but not general obesity is associated with poorer metabolic and cardiovascular health in premenopausal and postmenopausal Asian women. Maturitas.

[8] Gu Z, Zhu P, Wang Q, He H, Xu J, Zhang L, et al (Dis2018). Obesity and lipidrelated parameters for predicting metabolic syndrome in Chinese elderly population. Lipids Health.

[9] Li R, Li Q, Cui M, Yin Z, Li L, Zhong T, et al (2018). Clinical surrogate markers for predicting metabolic syndrome in middle-aged and elderly Chinese. J Diabetes Investig.

[10] Du T, Yuan G, Zhang M, Zhou X, Sun X, Yu X (2014). Clinical usefulness of lipid ratios, visceral adiposity indicators, and the triglycerides and glucose index as risk markers of insulin resistance. Cardiovasc Diabetol.

[11] Lee HJ, Jo HN, Kim YH, Kim SC, Joo JK, Lee KS (2018). Predictive value of lipid accumulation product, fatty liver index, visceral adiposity index for metabolic syndrome according to menopausal status. Metab Syndr Relat Disord.

[12] Du T, Yu X, Zhang J, Sun X (2015). Lipid accumulation product and visceral adiposity index are effective markers for identifying the metabolically obese normal-weight phenotype. Acta Diabetol.

[13] Hallajzadeh J, Khoramdad M, Izadi N, Karamzad N, Almasi- Hashiani A, Ayubi E, et al (2018). Metabolic syndrome and its components in premenopausal and postmenopausal women: a comprehensive systematic review and meta-analysis on observational studies. Menopause.

[14] Pu D, Tan R, Yu Q, Wu J (2017). Metabolic syndrome in menopause and associated factors: a meta-analysis. Climacteric.

[15] Grundy SM, Cleeman JI, Daniels SR, Donato KA, Eckel RH, Franklin BA, et al (2005). Diagnosis and management of the metabolic syndrome: an American Heart Association/National Heart, Lung, and Blood Institute Scientific Statement. Circulation.

[16] Simental-Mendia LE, Rodriguez-Moran M, Guerrero-Romero F (2008). The product of fasting glucose and triglycerides as surrogate for identifying insulin resistance in apparently healthy subjects. Metab Syndr Relat Disord.

[17] Amato MC, Giordano C, Galia M, Criscimanna A, Vitabile S, Midiri M, et al (2010). Visceral Adiposity Index: a reliable indicator of visceral fat function associated with cardiometabolic risk. Diabetes Care..

[18] Kahn HS (2005). The “lipid accumulation product” performs better than the body mass index for recognizing cardiovascular risk: a populationbased comparison. BMC Cardiovasc Disord.

[19] Wilson PW, D’Agostino RB, Parise H, Sullivan L, Meigs JB (2005). Metabolic syndrome as a precursor of cardiovascular disease and type 2 diabetes mellitus. Circulation.

[20] Carr MC (2003). The emergence of the metabolic syndrome with menopause. J Clin Endocrinol Metab.

[21] Rodrigues MH, Bruno AS, Nahas-Neto J, Santos ME, Nahas EA (2014). Nonalcoholic fatty liver disease and metabolic syndrome in postmenopausal women. Gynecol Endocrinol.

[22] Won KB, Park GM, Lee SE, Cho IJ, Kim HC, Lee BK, et al (2018). Relationship of insulin resistance estimated by triglyceride glucose index to arterial stiffness. Lipids Health Dis.

[23] Lambrinoudaki I, Kazani A, Armeni E, Rizos D, Augoulea A, Kaparos G, et al (2018). The metabolic syndrome is associated with carotid atherosclerosis and arterial stiffness in asymptomatic, nondiabetic postmenopausal women. Gynecol Endocrinol.

[24] Lambrinoudaki I, Kazani MV, Armeni E, Georgiopoulos G, Tampakis K, Rizos D, et al (2018). The TyG Index as a Marker of Subclinical Atherosclerosis and Arterial Stiffness in Lean and Overweight Postmenopausal Women. Heart Lung Circ.

[25] Maturana MA, Moreira RM, Spritzer PM (2011). Lipid accumulation product (LAP) is related to androgenicity and cardiovascular risk factors in postmenopausal women. Maturitas.

[26] Wehr E, Pilz S, Boehm BO, März W, Obermayer-Pietsch B (2011). The lipid accumulation product is associated with increased mortality in normal weight postmenopausal women. Obesity (Silver Spring).

[27] Lambrinoudaki I, Armeni E, Georgiopoulos G, Kazani M, Kouskouni E, Creatsa M, et al (2013). Subclinical atherosclerosis in menopausal women with low to medium calculated cardiovascular risk. Int J Cardiol.

